# Comparative Metabolomics and Network Pharmacology Study Reveals Chemopreventive Potential of Wild Soybean (*Glycine soja*)

**DOI:** 10.3390/foods15071209

**Published:** 2026-04-02

**Authors:** Meinan Sui, Zixin Yan, Long Xu, Dejiang Liu, Pengxia Zhang, Hong Zhao

**Affiliations:** 1 College of Biology and Agriculture, Jiamusi University, Jiamusi 154007, China; smnsoybean@163.com (M.S.); 15245692669@163.com (Z.Y.); jmsxl@126.com (L.X.); liudejiang2004@163.com (D.L.); 2 Heilongjiang Institute of Pharmacy, Jiamusi 154007, China; 3 Key Laboratory of Microecology-Immune Regulatory Network and Related Diseases, School of Basic Medicine, Jiamusi University, Jiamusi 154000, China; 4 College of Pharmacy, Jiamusi University, Jiamusi 154007, China

**Keywords:** soybean seeds, metabolic profiling, target network analysis, functional food, chemoprevention, bioactive compounds

## Abstract

Wild soybean (*Glycine soja*), as the wild ancestor of cultivated soybean, serves as a rich reservoir of phytochemicals with significant potential in functional food applications and chemoprevention. However, its metabolic characteristics and health benefits remain to be systematically elucidated. In this study, non-targeted metabolomics technology was employed, in conjunction with network pharmacology and molecular docking analysis, to systematically investigate the metabolic differences between wild soybean seeds from three distinct ecological regions and cultivated soybean seeds. Metabolomic profiling revealed the unique metabolic characteristics of wild soybean, identifying 124 significantly upregulated metabolites and 7 unique compounds, with the most notable enrichment in flavonoids and prunolides. Network pharmacology analysis indicated that 22 key metabolites in wild soybeans were associated with 503 pan-cancer targets (covering breast, lung, and colorectal cancers), primarily regulating pathways related to “cancer” and “lipids and atherosclerosis.” Molecular docking experiments further confirmed the stable binding affinity of key bioactive components, including quercetin and L-arginine, with core targets such as TP53, TNF, EGFR, IL1B, and JUN. These findings elucidate the unique phytochemical profile of wild soybean and its potential multi-target chemopreventive mechanisms, providing theoretical support for developing it as a natural chemopreventive agent.

## 1. Introduction

Soybean (*Glycine max*), as a globally significant oilseed and food crop, holds immense economic value and nutritional significance. Beyond its essential nutrients, soybeans are rich in diverse bioactive metabolites, including flavonoids, saponins, phytosterols, and lignans [1,2,3]. A wealth of evidence confirms that these phytochemicals possess multifaceted health-promoting properties, exhibiting antioxidant, anti-obesity, anti-tumor, and blood-sugar-lowering activities [4,5,6]. Consequently, exploring the bioactive potential of these soybean components is crucial for developing functional foods to prevent chronic diseases.

Plants, including soybean, exert their physiological functions through complex synergistic regulatory networks involving multiple targets and signaling pathways, differing from the single-target action mode of synthetic drugs. Previous studies have demonstrated that bioactive peptides in soybean exert anti-obesity effects by regulating key targets such as AKT1, SRC, and STAT3, thereby modulating the PI3K-Akt and JAK-STAT signaling cascades [4,6]. The chemical diversity of secondary metabolites provides the fundamental basis for this multi-target action mode. Furthermore, the widespread metabolic phenotypic heterogeneity within the genus *Glycine* constitutes a vital germplasm resource for identifying specific highly active functional components, opening up promising avenues for precision nutrition applications.

Among the abundant germplasm resources of the genus *Glycine*, wild soybean (*Glycine soja* Sieb.et Zucc.) serves as a vital genetic reservoir as the close ancestral relative of cultivated soybean (*Glycine max* L. Merr.). During long-term domestication, wild soybeans have retained key secondary metabolites that have significantly diminished or disappeared in cultivated varieties. Research indicates that the color phenotype of soybean seed coats is closely associated with metabolite accumulation (e.g., black-coated varieties are rich in anthocyanins). However, recent multi-omics analyses reveal that metabolic differences between wild and cultivated soybeans extend far beyond single pigment traits [7,8]. Unique flavonoids absent in 23 cultivated varieties accumulate in wild soybean seed coats [9]. A comparative metabolomics study based on multiple genotypes further confirmed extensive metabolic heterogeneity, involving nearly a hundred differentially expressed metabolites, including isoflavones, free amino acids, and fatty acids [10,11,12].

Of particular importance is the significant difference in secondary metabolite accumulation between wild and cultivated soybeans. These unique components primarily involve terpenoids, lipids, and flavonoids, which are not only crucial for plant defense and stress responses but also exhibit wide-ranging bioactivities, such as anti-inflammatory, antimicrobial, antitumor, and cardioprotective effects [13]. However, the existing research has primarily focused on the utilization of cultivated soybeans. The potential applications of these unique or highly abundant components in wild soybeans for chronic disease prevention remain to be thoroughly explored.

Breast cancer, lung cancer, and colorectal cancer rank among the three most prevalent malignant tumors globally, with substantial evidence indicating that their occurrence and progression are closely linked to dietary patterns [14,15]. The role of functional foods in cancer prevention has been gaining increasing attention. This study focuses on functional bioactive compounds in wild soybean seeds, aiming to explore their potential as chemopreventive dietary interventions against prevalent malignant tumors (e.g., breast, lung, and colorectal cancers). By integrating non-targeted metabolomics and network pharmacology techniques, we systematically compared the metabolic profiles of wild soybeans collected from three ecological regions with those of cultivated soybeans. Specifically, we identified characteristic differential metabolites that are significantly enriched in wild soybeans, predicted their corresponding pan-cancer targets and associated signaling pathways, and validated the binding affinities of key bioactive compounds with core cancer-related targets via molecular docking. The present work establishes a theoretical framework for the development of functional foods and natural chemopreventive agents, facilitating high-value utilization of wild soybean genetic stocks.

## 2. Materials and Methods

### 2.1. Plant Materials

Three wild soybean (*Glycine soja*, GS) accessions (GS-XK, GS-QF, and GS-SFS) and their corresponding local cultivated soybean (*Glycine max*, GM) varieties (GM-KF, GM-LK, and GM-KD94) were collected from three distinct ecological regions in Heilongjiang Province, China (Heihe, Fuyuan and Jiamusi, respectively). Mature seeds were harvested in October 2025. To account for biological variation, seeds from 10 randomly selected healthy plants were pooled to constitute a single biological replicate for each accession, with three independent biological replicates prepared in total. Upon collection, all samples were snap-frozen in liquid nitrogen and maintained at –80 °C until metabolite extraction.

### 2.2. Untargeted Metabolomic Profiling of Soybean Seeds

Untargeted metabolomic profiling was employed to comprehensively characterize the chemical composition of the soybean seeds [16]. Analyses were executed on a high-resolution liquid chromatography–mass spectrometry (LC-HRMS) system. This platform combined a Thermo Vanquish Flex UHPLC with an Orbitrap Exploris 120 mass spectrometer (Thermo Fisher Scientific, Waltham, MA, USA) [17]. Metabolite identification and relative quantification were carried out using MS-DIAL software (version 4.9.221218) [18].

#### 2.2.1. Sample Preparation

Briefly, 40 mg of ground soybean seed were blended with 300 μL of ice-cold methanol/acetonitrile/water (2:2:1, *v*/*v*) solution that included 5 ppm L-2-chlorophenylalanine acting as the internal standard. The resulting blend was ground using a high-throughput tissue grinder (Jingxin, Shanghai, China) (55 Hz, 60 s, repeated once), followed by ultrasonic extraction for a period of 10 min. After incubation at −20 °C over a span of 30 min to precipitate proteins, the extract was centrifuged at 12,000 rpm for a period of 10 min at 4 °C. The supernatant obtained from the 2 μL sample was analyzed by LC-MS after being filtered through a 0.22-μm membrane. Quality control (QC) samples were generated by mixing equivalent portions of all experimental samples, and were injected at regular intervals during the entire analytical process to evaluate instrument stability and validate data reproducibility.

#### 2.2.2. Chromatography and Mass Spectrometry Parameters

Analytes were separated on an ACQUITY UPLC HSS T3 column (100 Å, 1.8 μm, 2.1 × 100 mm) held at 40 °C (flow rate 0.4 mL/min). The mobile phases were water (A) and acetonitrile (B), each containing 0.1% formic acid, with the following gradients: 0–1 min, 5% B; 1–4.7 min, linear ramp to 95% B; 4.7–6 min, hold at 95% B; and 6.1–8.5 min, re-equilibration at 5% B, respectively. This short gradient was validated in prior untargeted metabolomics work and ensured adequate resolution and metabolite coverage [19,20].

MS data were collected via data-dependent acquisition (DDA) in positive/negative ionization modes (scan range 70–1000 *m*/*z*). HESI source settings were tuned as follows: spray voltage +3.5 kV (+)/−3.0 kV (−); sheath gas 40, auxiliary gas 10 (arbitrary units); capillary temp 320 °C; auxiliary gas heater temp 300 °C. Full MS scans were acquired at 60,000 resolution. For MS/MS, the top 4 most abundant ions were fragmented by higher-energy collisional dissociation (HCD) with 30% normalized collision energy (NCE) at 15,000 resolution.

#### 2.2.3. Data Processing and Quantification

Raw data were processed using MS-DIAL software to perform peak extraction, filtering, metabolite identification, and other operations. The missing value imputation algorithm was used to impute missing values for undetected peaks, and compounds with a QC RSD > 30% were filtered out. Metabolite identification was performed using PerSonalbio’s PSNGM (PerSonalbio Next-Generation Metabolomics Database). This database includes a self-built standard library, mzCloud (https://www.mzcloud.org/), HMDB (https://hmdb.ca/), MoNA (https://mona.fiehnlab.ucdavis.edu), NIST_2020_MSMS, and AI-predicted MS/MS spectrum libraries. The key parameters were as follows: MS1 tolerance for identification 0.01; MS2 tolerance for identification 0.05; smoothing level 3; minimum peak height 10,000; minimum peak width 5; mass slice width 0.05; and identification score cutoff 70.

#### 2.2.4. Data Analysis

All experiments were carried out in triplicate with three independent biological replicates. Multivariate statistical analysis, including Principal Component Analysis (PCA) and Partial Least Squares Discriminant Analysis (PLS-DA), were performed using the ropls package (v1.22.0) in R. Differential metabolites were screened by combining a Variable Importance in Projection (VIP) score > 1 (obtained from the PLS-DA model) and a *p*-value < 0.05 (calculated via Student’s *t*-test). Permutation testing with 100 iterations was performed to validate model robustness and rule out overfitting.

### 2.3. Network Pharmacology Analysis Methods

#### 2.3.1. Metabolite and Disease Target Identification

Potential protein targets of the differential metabolites were forecast using the TCMSP database (https://www.tcmsp-e.com/) and the STITCH database (http://stitch.embl.de/). Retrieved target proteins were mapped to official gene symbols through the UniProt database (https://www.uniprot.org/), with the species limited to *Homo sapiens*.

Disease-associated targets for breast cancer, lung cancer, and colorectal cancer were obtained from the GeneCards, OMIM, and TTD databases. To identify targets associated with broad-spectrum anticancer activity, separate target libraries were first constructed for each cancer type by merging the targets obtained from these three databases. Subsequently, the intersection of these three independent libraries was calculated to yield common disease targets (pan-cancer targets). These common targets were then intersected with the predicted metabolite targets. The overlapping targets were visualized via Venn plots, which were created using the VennDiagram package (version 1.7.3) in the R environment. The identified shared targets were subsequently utilized for “metabolite–target–disease” network construction and enrichment analysis.

#### 2.3.2. Construction of Protein Interaction Networks and Metabolite–Target–Disease Networks

To characterize the interactions among target proteins, the overlapping targets were uploaded to the STRING database (https://string-db.org/), with the species limited to *Homo sapiens* and a minimum interaction confidence score of 0.7. The resulting protein–protein interaction (PPI) data were visualized and analyzed using Cytoscape 3.9.1. Node degree centrality was calculated using the igraph package (v2.2.1) in R to identify core targets based on their topological importance.

A “metabolite–target–disease” network was constructed by integrating the differential metabolites and the shared disease targets in Cytoscape 3.9.1. In this network, nodes represent metabolites, targets, or diseases, while edges denote their interactions. Key metabolites and core targets were identified through network topological analysis, prioritizing nodes with higher degree values.

#### 2.3.3. GO and KEGG Pathway Enrichment Analysis

For the purpose of exploring the biological functions and signaling pathways associated with the shared target genes, Gene Ontology (GO) and Kyoto Encyclopedia of Genes and Genomes (KEGG) enrichment analyses were carried out in R with the clusterProfiler package (version 4.14.6). The hypergeometric test was applied to calculate raw *p*-values, and the Benjamini–Hochberg (BH) approach was utilized for multiple testing correction. GO terms and KEGG pathways with an adjusted *p*-value < 0.01 were identified as statistically significant, and all results were ordered from the smallest to the largest adjusted *p*-value.

### 2.4. Molecular Docking

Three-dimensional structural models of the core target proteins were downloaded from the PDB database (https://www.rcsb.org/). Missing residues and side chains were repaired, and water molecules were removed using PDBFixer 1.12, followed by the addition of polar hydrogen atoms. Differential metabolites were prepared using OpenBabel 3.1.1 for format conversion, hydrogenation, and energy minimization. Molecular docking simulations were performed using AutoDock Vina 1.2.7. The optimal binding conformation for each metabolite–target pair was selected based on the lowest binding energy (affinity, kcal/mol) and the docking results were visualized and analyzed using PyMOL 2.6.2 [21,22].

## 3. Results

### 3.1. Metabolomics Data Quality Assessment and Sample Metabolic Profile Characteristics

To elucidate the differences in metabolic composition between wild and cultivated soybean germplasm, we employed a non-targeted metabolomics strategy based on LC-HRMS. A paired sampling design was adopted, collecting three wild soybean accessions (GS-XK, GS-QF, GS-SFS) and their corresponding local cultivated varieties (GM-KF, GM-LK, GM-KD94) from three distinct ecological zones in Heilongjiang Province. A total of 18 samples were analyzed.

**Figure 1:**
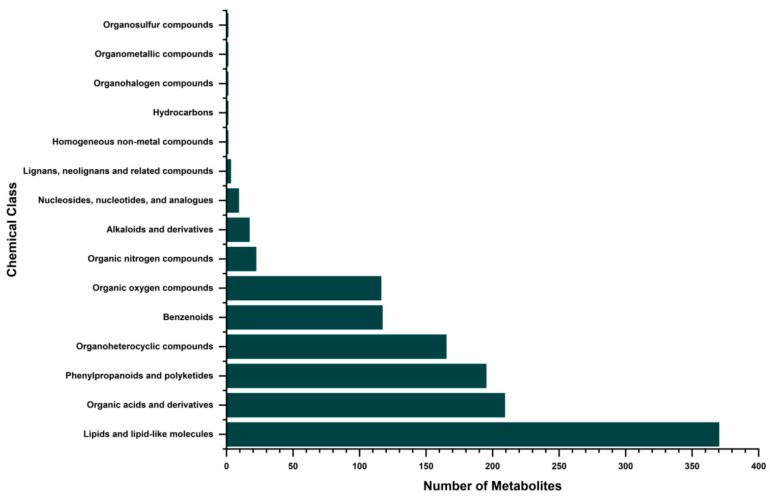
Chemical classification of metabolites detected in wild and cultivated soybean seeds.

The platform detected 1281 metabolites (689 in positive ion mode and 592 in negative ion mode). Among these, 1228 metabolites (96%) were assigned with functional annotations and classified into 15 chemical categories (Figure 1). The primary categories included lipids and lipid-like molecules, organic acids and derivatives, and phenylpropanoids and polyketides. To guarantee data quality, QC samples were analyzed. The results showed that over 65% of the metabolite peaks had a relative standard deviation (RSD) ≤ 15%, and 80% had an RSD ≤ 20% (Figure S1), confirming the robustness of the dataset.

### 3.2. Multivariate Statistical Analysis of Metabolic Profiles

**Figure 2:**
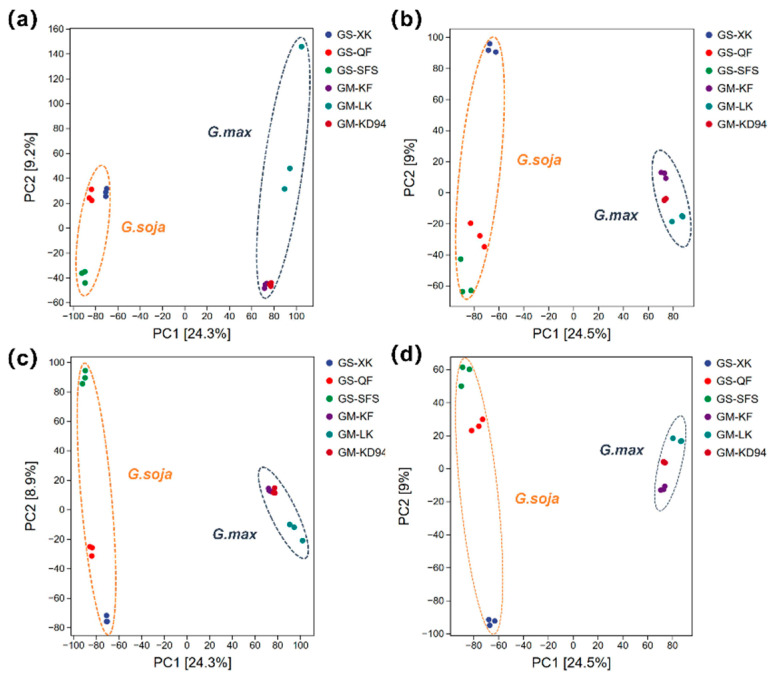
Score plots of PCA and multi-group PLS-DA for wild soybean (*G. soja*: GS-XK, GS-QF, GS-SFS) and cultivated soybean (*G. max*: GM-KF, GM-LK, GM-KD94). (**a**) PCA in positive ion mode; (**b**) PCA in negative ion mode; (**c**) multi-group PLS-DA in positive ion mode; (**d**) multi-group PLS-DA in negative ion mode.

PCA was performed to evaluate global metabolic variation. In both ion modes, the PCA score plots indicated a tendency of separation between wild and cultivated soybeans along the PC1 axis (Figure 2a,b). PC1 explained 24.3% (positive) and 24.5% (negative) of the total variance, respectively.

Supervised pairwise PLS-DA models were subsequently established to screen for putative differentiating metabolites. The models showed a certain degree of separation trend, with RX^2^ values of 0.331 (positive ion mode) and 0.324 (negative ion mode), and Q^2^ values of 0.982 and 0.98, respectively. Permutation tests with 100 iterations suggested no obvious overfitting (Figure S2). Meanwhile, exploratory multi-group PLS-DA showed that cultivated soybean samples from different regions clustered closely along PC2, whereas wild soybean samples displayed a trend of separation corresponding to their ecological zones (Figure 2c,d), which may preliminarily suggest greater metabolic plasticity in wild populations.

### 3.3. Identification and Characterization of Differential Metabolites

**Figure 3:**
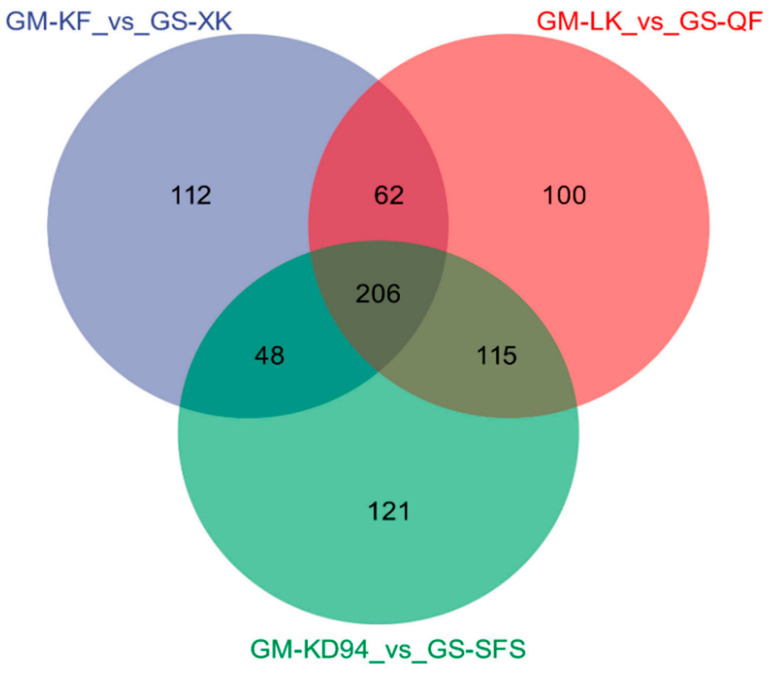
Venn diagram of differential metabolites in three pairwise comparisons between wild and cultivated soybean groups.

Differential metabolites were screened based on VIP > 1 and *p* < 0.05. In total, 764 differential metabolites were putatively annotated using high-resolution LC-MS/MS analysis, with structural identification supported by matching accurate mass, retention time, and characteristic MS/MS fragmentation patterns against in-house and public metabolite databases. Among them, 206 metabolites were stably detected across all experimental groups (Figure 3). Specifically, 124 metabolites were significantly upregulated in wild soybean (fold change (FC) > 1.5, *p* < 0.05) (Table S1). These upregulated metabolites were classified into 27 categories, with flavonoids showing the highest enrichment (20.16%), followed by prenol lipids (17.74%) and benzene-substituted derivatives (12.10%).

Furthermore, seven metabolites were detected exclusively in wild soybean seeds across all three ecological regions and were absent in all cultivated varieties. These wild-specific metabolites included five flavonoids, one prenol lipid, and one glycerophospholipid. Additionally, we identified ten metabolites present in wild soybeans across all ecological regions but detected in cultivated soybeans from only one of the three ecological regions (Table S2).

### 3.4. Network Pharmacology Analysis

#### 3.4.1. Screening of Potential Therapeutic Targets

**Figure 4:**
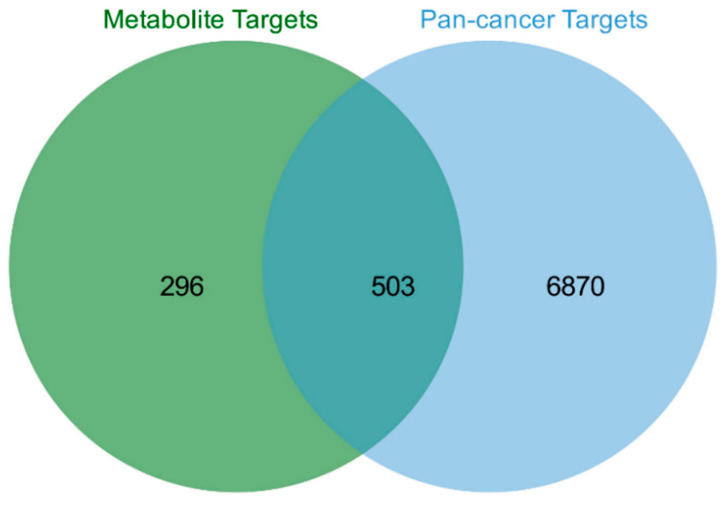
Venn diagram showing overlapping targets between key differential metabolites and pan-cancer disease targets.

To explore the potential chemopreventive mechanisms, target prediction was performed for the 124 upregulated and 7 wild-specific metabolites. Among them, 44 HMDB-annotated metabolites possessed documented target information in the TCMSP and STITCH databases, identifying 799 unique candidate targets associated with 23 metabolites. Concurrently, 7373 consensus targets associated with breast, lung, and colorectal cancers were obtained from the GeneCards, OMIM, and TTD databases. Intersection analysis yielded 503 potential targets corresponding to 22 bioactive metabolites (Figure 4, Table S3).

#### 3.4.2. PPI Network and “Metabolite–Target–Disease” Network Construction

**Figure 5:**
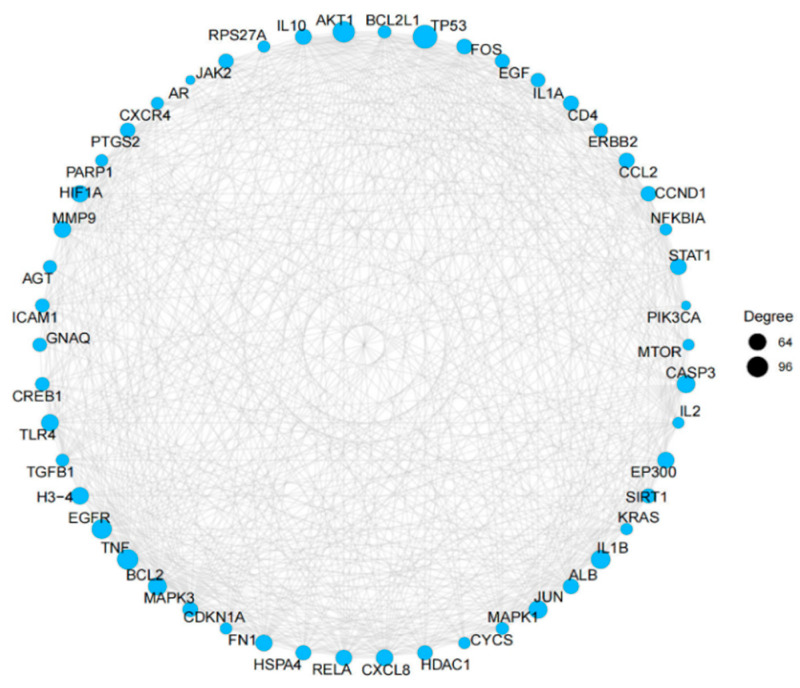
PPI network diagram of 503 intersecting pan-cancer targets (lung cancer, breast cancer, and colorectal cancer).

To explore the interaction profiles of the 503 potential pan-cancer targets and identify key targets, we constructed a PPI network consisting of 451 nodes and 3461 edges (Figure 5). Topological analysis identified the top five hub genes: *TP53*, *TNF*, *EGFR*, *IL1B*, and *JUN*.

**Figure 6:**
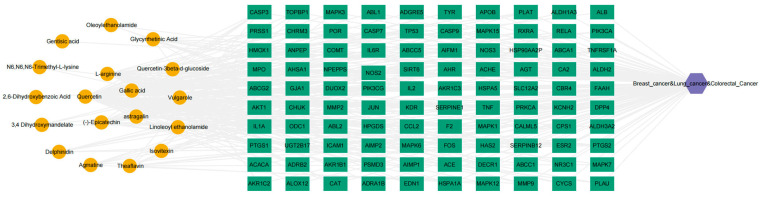
Metabolite–target–disease network diagram based on key differential metabolites and top 100 hub pan-cancer targets.

Subsequently, a “metabolite–target–disease” network was constructed, and the top 100 targets ranked by degree were prioritized for visualization (Figure 6). Notably, these hub targets were found to interact with 18 of the 22 metabolites. Importantly, the top five metabolites in Figure 6 are ranked based on their degree within the subnetwork (i.e., the top 100 targets). In the overall network encompassing all 503 targets, quercetin (degree = 261) and L-arginine (degree = 176) exhibited the highest connectivity, indicating their central regulatory role, followed by N6,N6,N6-trimethyl-L-lysine, (-)-epicatechin, and glycyrrhetinic acid.

#### 3.4.3. GO Functional Enrichment Analysis and KEGG Pathway Analysis

**Figure 7:**
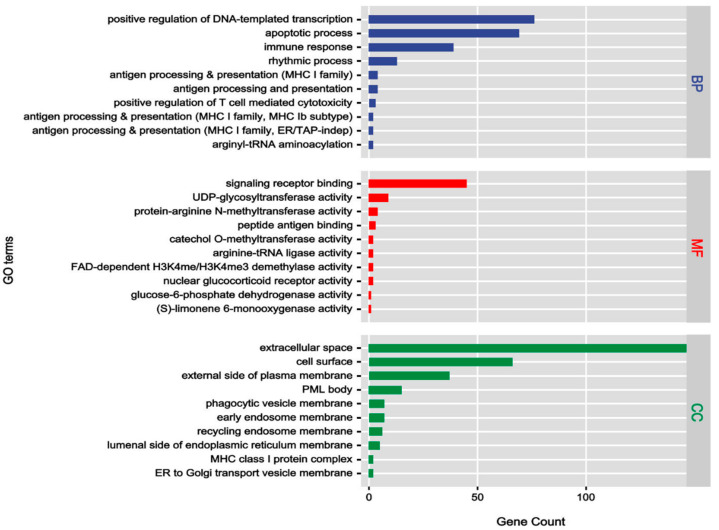
GO enrichment analysis results of intersecting pan-cancer targets.

GO enrichment analysis identified 183 significant entries (*p* < 0.01), categorized into 95 biological processes (BPs), 61 molecular functions (MFs), and 27 cellular components (CCs) (Table S4). As shown in Figure 7, the BPs were primarily enriched in positive regulation of DNA-templated transcription, apoptotic process, and immune response. Regarding MF, the entries were dominated by signaling receptor binding, UDP-glycosyltransferase activity, and protein arginine N-methyltransferase activity. As for CC, targets were predominantly localized in the extracellular space and plasma membrane.

**Figure 8:**
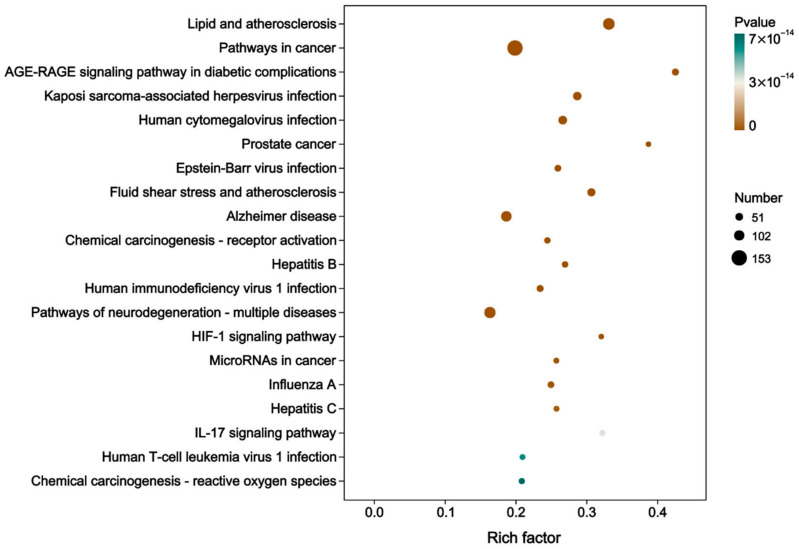
Top 20 pathways from KEGG pathway enrichment analysis of intersecting pan-cancer targets.

KEGG pathway analysis revealed 399 targets significantly enriched across 154 pathways (Table S5). “Pathways in cancer” (hsa05200) showed the highest enrichment, followed by “Lipids and atherosclerosis” (hsa05417) and “Neurodegeneration—multiple diseases” (hsa05022) (Figure 8).

### 3.5. Molecular Docking Validation

**Figure 9:**
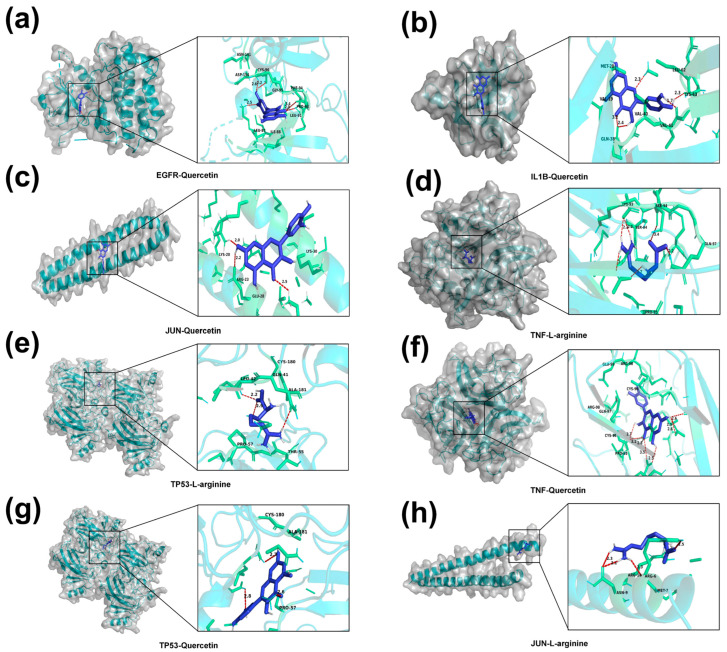
Global and local molecular docking analysis of interactions between key compounds (quercetin and L-arginine) and core targets (TP53, TNF, EGFR, IL1B, JUN). (**a**) EGFR-quercetin; (**b**) IL1B-quercetin; (**c**) JUN-quercetin; (**d**) TNF-L-arginine; (**e**)TP53-L-arginine; (**f**) TNF-quercetin; (**g**) TP53-quercetin; (**h**) JUN-L-arginine.

Molecular docking was performed to assess the binding affinity between key metabolites (quercetin and L-arginine) and core targets (TP53, TNF, EGFR, IL1B, JUN) (Figure 9). The docking results demonstrated that all tested pairs exhibited binding energies below −1.2 kcal/mol. Notably, the binding energies of glycyrrhetinic acid with EGFR, IL1B, JUN, TP53 and TNF, as well as those of L-arginine with TNF and TP53, were all less than −5.0 kcal/mol (Table S6), indicating stable binding conformations.

## 4. Discussion

Wild soybeans represent a vital genetic resource repository, yet the domestication process has diminished their genetic diversity, reduced metabolite production, and lowered stress resistance [23]. Our exploratory results suggest a tendency of metabolic divergence between wild and cultivated soybeans. This phenomenon may primarily stem from genetic background differences rather than environmental factors. These intrinsic metabolic variations could provide a useful basis for subsequent identification and functional analysis of differential metabolites.

The 124 differential metabolites detected with higher content in wild soybean seeds than cultivated soybeans primarily included flavonoids (20.16%), prenol lipids (17.74%), and benzene-substituted derivatives (12.10%). They enhance plant stress resistance and their value as functional food components is particularly prominent. Flavonoids and prenol lipids are widely recognized as bioactive components with potential chemopreventive properties [24,25], while benzene-substituted derivatives, such as gallic acid and gentisic acid, have demonstrated efficacy in reducing risks of breast and colorectal cancers [26,27]. Furthermore, the identification of seven wild-specific metabolites (predominantly flavonoids) in wild soybeans reveals metabolic characteristics that have been progressively weakened or lost in cultivated varieties, highlighting the potential of wild soybeans as a valuable genetic resource for developing functional foods and nutritional supplements.

To further explore the potential value of metabolites significantly enriched in wild soybean, we predicted their antitumor effects. Given that breast cancer, lung cancer, and colorectal cancer are the three most prevalent cancers globally, identifying their common targets could provide a crucial pathway for developing broad-spectrum cancer therapies. To this end, we conducted network pharmacology analysis on 44 upregulated metabolites with available HMDB IDs and known targets. In contrast, seven wild-type-specific metabolites and the remaining upregulated metabolites were excluded from this analysis due to incomplete structural identification or a lack of target data. They represent potential targets for future structural elucidation and bioactivity validation.

The results of this study show that 503 potential targets corresponding to 22 bioactive metabolites were identified. Five hub targets (TP53, TNF, EGFR, IL1B, and JUN) were discovered in the network diagram as the core nodes potentially linked to the shared mechanisms of breast, lung, and colorectal cancers. Specifically, TP53 and EGFR are pivotal in regulating cell cycle arrest and malignant progression [28,29], while TNF and IL1B bridge chronic inflammation and tumor immune evasion [30,31]. JUN, which is involved in cell proliferation and metastasis, further highlights the multi-faceted regulatory potential of these metabolites [32]. The enrichment of these genes in the PPI network highlights their potential as broad-spectrum therapeutic targets.

Further elucidation of the “metabolite–target–disease” network revealed that quercetin and L-arginine are the most central bioactive compounds. Notably, quercetin, a flavonoid abundant in soybeans, emerged as a key regulator capable of simultaneously targeting TP53 and EGFR [33], consistent with its recognized antioxidant and anticancer properties. Similarly, L-arginine exerts multifaceted roles in tumor metabolism and immune regulation [34,35,36]. The high connectivity of these metabolites suggests they may function as core regulators, exerting pleiotropic effects by modulating multiple pivotal targets.

GO and KEGG enrichment analyses elucidated the biological significance of the target genes. The GO analysis revealed that biological processes were primarily enriched in DNA-templated transcription, apoptotic processes, and immune response. The enrichment of apoptotic processes underscores their critical role in eliminating malignant cells [37]. Meanwhile, the immune response terms suggest that these components may modulate the tumor microenvironment to reactivate immune surveillance and overcome immune escape [38]. KEGG pathway analysis further supported these findings, showing significant enrichment in “Pathways in cancer”. Interestingly, the “Lipids and atherosclerosis” and “Neurodegeneration” pathways were also significantly enriched, suggesting that the active components may affect fundamental cellular pathways, such as oxidative stress and apoptosis, which are common to both cancer and neurodegeneration.

Finally, molecular docking was employed to validate the binding affinity between the key metabolites (quercetin and L-arginine) and core targets. The results indicated stable binding conformations, with most compounds exhibiting favorable binding energies. Specifically, the strong affinity of quercetin for targets such as EGFR and IL1B corroborates previous reports of its direct inhibitory effects on these proteins [39,40,41,42,43]. For L-arginine, the observed binding potential with TNF and TP53 aligns with its biological role in inflammation and cell survival pathways, where it serves as a critical substrate [35]. These findings provide structural evidence supporting the reliability of the network pharmacology predictions.

## 5. Conclusions

This study demonstrates that wild soybean (*Glycine soja*) seeds exhibit significantly superior metabolic characteristics compared to cultivated varieties. Through a strategy combining non-targeted metabolomics and network pharmacology, we comprehensively characterized the metabolite profiles of wild soybean and identified a rich array of functional metabolites. Among them, quercetin and L-arginine were structurally identified with high confidence based on accurate mass-to-charge ratio (*m*/*z*) matching, retention time alignment, and diagnostic MS/MS fragmentation patterns, which were compared against authentic standard references and public spectral databases. These core metabolites demonstrate potential for regulating broad-spectrum antitumor effects by targeting key regulatory hubs, including TP53, TNF, EGFR, IL1B, and JUN. These findings were further validated through molecular docking simulations. This study bridges the gap between wild soybean resource development and functional food innovation, establishing a robust molecular foundation for future nutritional supplement research and development.

## Data Availability

The original contributions presented in the study are included in the article/Supplementary Materials. Further inquiries can be directed to the corresponding authors.

## Supplementary Materials

The following supporting information can be downloaded at https://www.mdpi.com/article/10.3390/foods15071209/s1, Figure S1: Cumulative distribution of relative standard deviation (RSD) of metabolites in pooled quality control (QC) samples. (a) Positive ion mode; (b) negative ion mode. The purple and green lines indicate the 15% and 20% RSD thresholds, respectively; Figure S2: Permutation test results for PLS-DA model reliability with 100 iterations. (a) Positive ion mode; (b) negative ion mode. Table S1: The 124 significantly upregulated metabolites in wild soybeans compared with cultivated soybeans (fold change (FC) > 1.5, *p* < 0.05); Table S2: Information of metabolites widely distributed in wild soybean but restricted in cultivated soybean. Table S3: The 503 potential targets of bioactive metabolites with chemopreventive effects against pan-cancer; Table S4: GO enrichment analysis of the 503 potential targets of bioactive metabolites against pan-cancer (*p* < 0.01); Table S5; KEGG enrichment analysis of the 503 potential targets of bioactive metabolites against pan-cancer (*p* < 0.01); Table S6: Binding energies of key anti-cancer metabolites and core targets.
