# Supplementary figures and images for: Comparative Metabolomics and Network Pharmacology Study Reveals Chemopreventive Potential of Wild Soybean (Glycine soja)
Source: Foods. 2026 Apr 2;15(7):1209. doi: 10.3390/foods15071209 (PMC13073708; doi:10.3390/foods15071209)

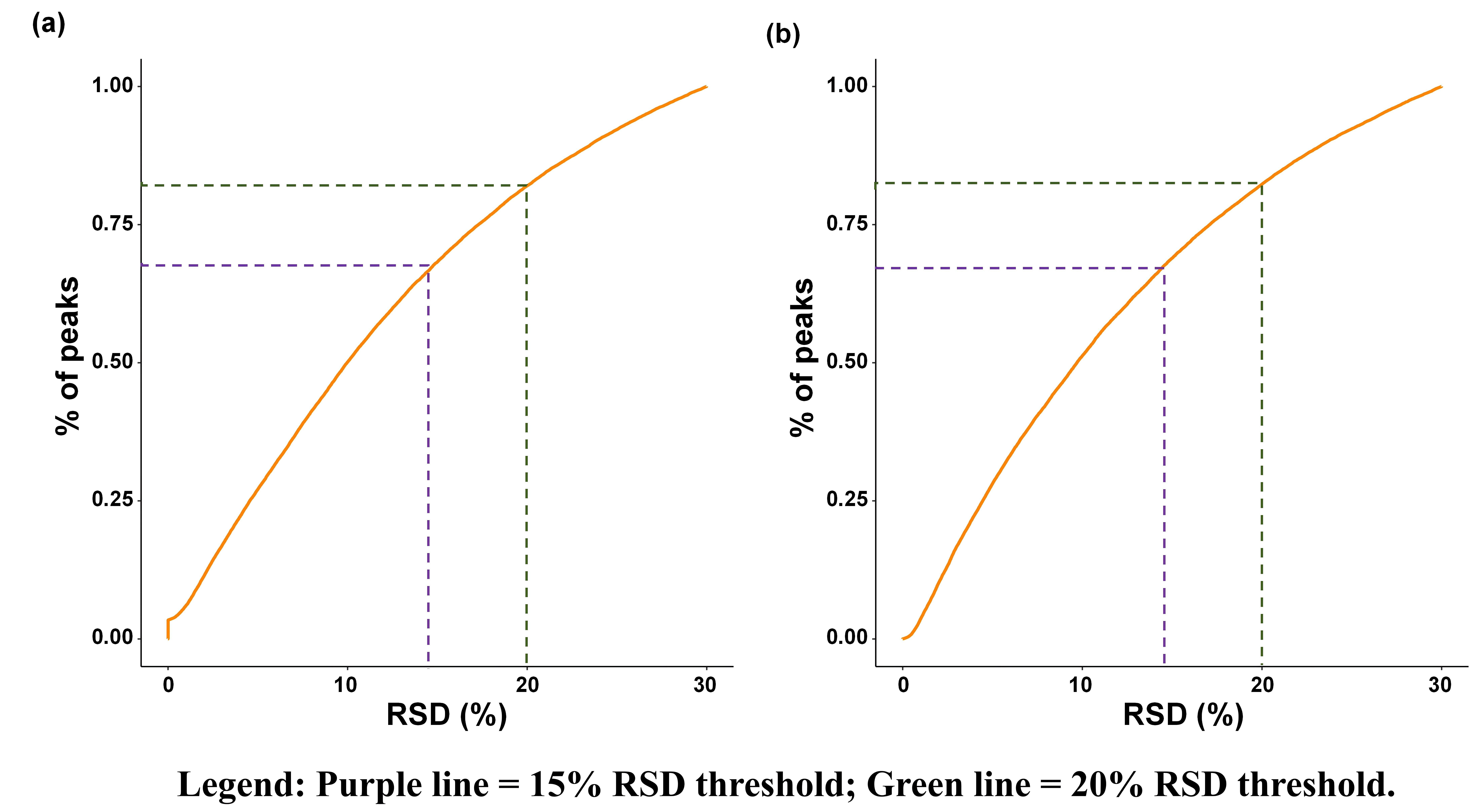

Supplement: Supplementary file 1 [file foods-15-01209-s001.zip › Figure S1.tiff]

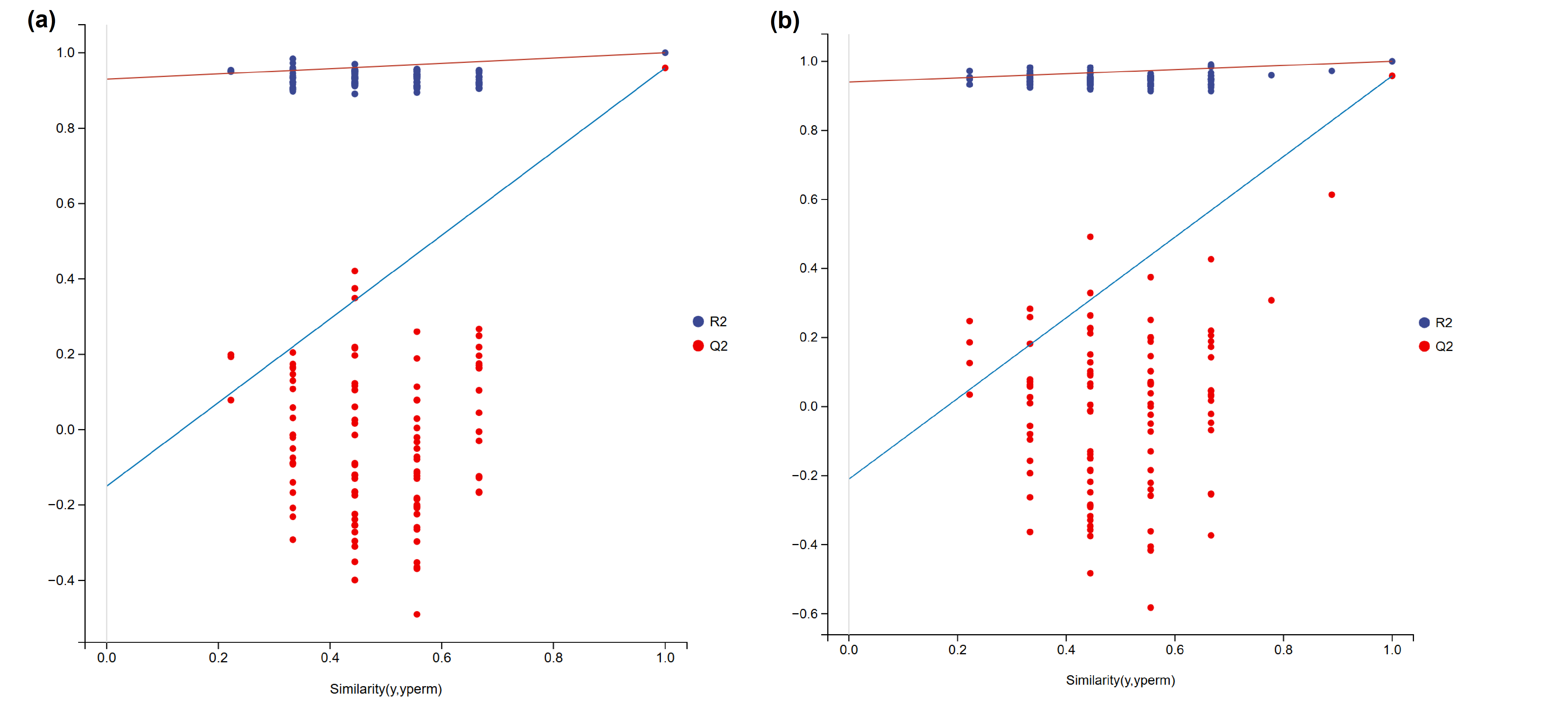

Supplement: Supplementary file 1 [file foods-15-01209-s001.zip › Figure S2.tiff]
